# Virtual Screening Approaches towards the Discovery of Toll-Like Receptor Modulators

**DOI:** 10.3390/ijms17091508

**Published:** 2016-09-09

**Authors:** Lucía Pérez-Regidor, Malik Zarioh, Laura Ortega, Sonsoles Martín-Santamaría

**Affiliations:** Department of Chemical & Physical Biology, Centro de Investigaciones Biológicas, CIB-CSIC, C/Ramiro de Maeztu, 9, 28040 Madrid, Spain; lucia.perez.regidor@cib.csic.es (L.P.-R.); m.zarioh17@gmail.com (M.Z.); l.ortegavarga@gmail.com (L.O.)

**Keywords:** Toll-like receptor, TLR modulators, virtual screening, drug discovery, computational approaches

## Abstract

This review aims to summarize the latest efforts performed in the search for novel chemical entities such as Toll-like receptor (TLR) modulators by means of virtual screening techniques. This is an emergent research field with only very recent (and successful) contributions. Identification of drug-like molecules with potential therapeutic applications for the treatment of a variety of TLR-regulated diseases has attracted considerable interest due to the clinical potential. Additionally, the virtual screening databases and computational tools employed have been overviewed in a descriptive way, widening the scope for researchers interested in the field.

## 1. Introduction

Innate immunity is the first defensive wall a pathogen needs to beat to flourish in our body and, due to the enormous diversity of microorganisms present in our environment, it is composed by a number of agents able to respond in a highly effective way. Among them, the Toll-like receptors (TLR) family can recognize a wide variety of pathogens, making them an interesting target to help our body fight disease [1]. Each TLR is specialized in the recognition of a particular pathogen-associated molecular pattern (PAMP) arisen from a bacteria or virus. TLRs 1, 2, 4, 5, and 6 are situated primarily in the plasma membrane, where they recognize bacterial components of microbial cell walls and membranes, such as lipopolysaccharide (LPS) and lipoteichoic acid from the cell wall, lipoproteins from the cell membrane, and flagellin. TLRs 3, 7, 8, and 9 are intracellularly located in the membranes of endosomes and lysosomes, where they bind to microbial nucleic acids, including double- and single-stranded RNA (dsRNA, ssRNA) from RNA viruses, and DNA from most organisms, including self-nucleic acids from the host cell [2].

TLR modulators have the potential to be used with different biomedical applications, especially in the field of infection [3], inflammation [4] and autoimmune diseases [5], but also in central nervous system (CNS) disorders such as Alzheimer´s disease [6], and cancer [7,8]. However, just a few of them are currently under clinical development. Therefore, it is imperative to find new chemical entities as TLR modulators with drug-like properties in order to facilitate their development as drugs. In the context of drug discovery, virtual screening (VS) techniques have already proved to make hit identification more goal-oriented, allowing the access to a huge number of chemically diverse binders (from public and commercial databases) with a relatively low-cost in terms of time and materials. This computational approach has been subjected to extensive attention and revision over the years, from the early perspective of being an emerging method [9], until the current time where new challenges are faced [10,11,12,13,14,15]. We could say that TLRs are not standard receptors which could be approached following classical strategies in drug design. The complexity of the system and the characteristics of their complexation with the PAMPs make them especially difficult to tackle following classical procedures in drug design and discovery. This is why TLRs constitute a special case study in this context. We herein report successful cases of VS approaches that have led to TLR modulators either with agonist or antagonist activity.

## 2. Databases

A wide variety of databases containing lead and drug-like small molecules is available for VS purposes which, inevitably, overlap. Several analysis and comparison studies among the available compound libraries have been carried out to evaluate molecular uniqueness and database overlapping, drug-like properties and scaffold diversity [16,17,18]. Overall, although a substantial overlapping is found among some of the collections, each database has unique features that may make them more adequate for a particular VS project, and still a relevant number of unique compounds is found within each database that makes it worth considering more than one library if possible. Duplicate analysis of screening libraries comprising the ones cited below showed that 40%–50% of the totality of structure subjected to analysis were structures exclusive to one supplier library [19,20]. Many of them are freely available and may possess desirable characteristics such as “drug-likeness”, being the most popular the “Lipinski Rule of Five” [21]. Others collect chemical structures from natural products or approved drugs [13]. We here report the databases that have been reported for VS approaches focused on Toll-like receptors.

### 2.1. ZINC Database

ZINC (recursive acronym for Zinc Is Not Commercial) is a public access database of commercially available compounds developed in the Department of Pharmaceutical Chemistry at the University of California, San Francisco [22]. It contains a constantly growing number of three-dimensional (3D) structures ready-to-dock from catalogues of major compound vendors with annotated relevant protonation and tautomeric states, and properties such as size, calculated logP, number of rotatable bonds, etc. Each molecule also contains purchasability and vendor information [23,24], making this ZINC’s focus on docking and purchasability the main distinctive characteristic from other databases. In its latest version, ZINC 15 [25] comprises over 120 million purchasable “drug-like” compounds together with information regarding target and biological activity, related scaffolds and bioactive and biogenic compounds (Tanimoto index of 0.6) [25,26,27]. It also offers other features such as the possibility to define target-focused libraries and to download subsets of a physical property space (“fragment-like”, lead-like, drug like subsets).

### 2.2. NCI Open Database

NCI Open Database is a freely accessible database developed by the Developmental Therapeutics Program of the National Cancer Institute [28] currently containing >250,000 molecules both from organic synthesis and natural source extracts [29] that can be downloaded in SDF format. Compounds can be requested with no fee for research purposes. This database contains a set of compounds that have been collected by the National Cancer Institute, NIH since 1955, in Human Tumor Cell Line Screens and from the 1980s in AIDS Antiviral Screen that are not covered due to a confidentiality agreement. The database contains information about release, structure source and evaluation, calculated/predicted logP, biological activity, commercial availability, 3D atom coordinates, added hydrogens and also number of rotatable bonds, stereocenters and bond stereocenters. It provides structures that cannot be found in any other databases [17].

### 2.3. ASINEX Database

ASINEX database [30] is a regularly updated commercial collection of compounds which contains to date: 600,000 screening compounds, 27,000 macrocycles, 23,000 fragments and 7000 building blocks. The different libraries cover different chemical characteristics and try to address different steps in the drug discovery process [23]. The broadest collection (Gold collection, 250,000 compounds) offers high diversity and drug-like space coverage; other libraries are focused on lead-like compounds (Platinum, in-house collection of 150,000 compounds) and drug-like compounds (ASINEX Synergy, 35,000 compounds) as well as novel scaffolds intended for early stages of drug discovery (Elite libraries, >900 scaffolds profiled to elude ADMET problems). A fragment set of more than 22,500 compounds and a natural product-based library, with key structural features of known pharmacologically relevant natural products (BioDesign, around 13,000 compounds), are also available. In addition, ASINEX offers targeted libraries (CNS-focused, immuno-oncology-focused, PPIs [31], GPCR, ion channels, kinases, peptide-mimetics, nucleoside-mimetics, glyco-mimetics, covalent inhibitors, antiviral, carbohydrates, etc.) and customized screening sets. All the collections can be downloaded in SDF format and they could be directly purchased.

### 2.4. SPECS Database

SPECS repository [32] (>240,000 compounds) is composed of novel drug-like small molecules obtained from academia and research institutes. SPECS’ stock is updated every month and it contains available compounds that can be purchased upon request. Every molecule in the collection must fulfill structural characteristics of a biologically active compound, and meet ADMET requirements. Furthermore, SPECS offers targeted libraries which can be generated by two methods. The first one analyses the stock database with an in-house predictive software to generate the collections based on the predicted biological activities (kinase inhibitors, protease inhibitors, signal pathway modulators, ion channel blockers, GPCR’s ligands, nuclear receptors modulators, antimicrobials, antivirals, cytostatics, central nervous system ligands). These focused libraries are generated from predictions based on 2D descriptors and chemical structures of known active compounds, also with a training set of more than 40,000 chemical structures with confirmed activity for more than 600 specified activities and therapeutic areas. The second method clusters within the SPECS’ chemical space, or rather descriptor space (accounting for topological and connectivity information, hydrophobic and hydrophilic effects, polarizability, and electrostatic interactions). This follows a principal component analysis and compounds are thus clustered together with hit molecules with reported biological activity, or from diverse commercial libraries.

### 2.5. MAYBRIDGE Database

Maybridge [33] Screening Hit Discovery collection (over 53,000 compounds) is a commercial library of small hit-like and lead-like organic compounds of high diversity (Tanimoto Clustering at 0.9) [34], that covers ca. 87% of the 400,000 theoretical drug pharmacophores with general compliance with the Lipinsky rule of five and of good ADMET properties. The HitCreator^TM^ Collection (selection of 14,400 of Maybridge screening compounds) aims to represent the diversity of the main collection covering the drug-like chemical space. Maybridge also offers a fragment library (30,000 fragments), a hit-to-lead building block collection, and a Ro3 2500 diversity fragment library (2500 fragments) with a Tanimoto similarity index of 0.66 (based on standard Daylight fingerprinting), assured solubility, optimized for SPR and Ro3 compliant. It provides special collections of Fluoro, ^19^Fluoro and Bromo-fragment libraries.

### 2.6. LIFE CHEMICALS Database

LIFE CHEMICALS [35] holds a commercial compound collection for High Throughput Screening (HTS) of 1,213,000 (431,000 in stock from distinctly different 2800 scaffolds) lead-like and drug-like new chemical entities selected taking into account diversity, Lipinski’s rules [21] and Veber criteria [36]. Furthermore, it offers several different diversity libraries on demand: building blocks, fragment- and scaffold-based libraries and different kinds of targeted/focused libraries (natural product-like compounds, covalent inhibitors, epigenetics-related compounds, PPI inhibitors, transmembrane receptors binders, nuclear receptor modulators, transporters, enzymes targeted compounds, etc.) [37,38,39]. The collection is available both as MDL SD (.sdf) or MDL ISIS (.db) files.

### 2.7. ENAMINE Database

ENAMINE [40] provides several different commercial collections of compounds for screening: the HTS collection (>1,720,000 compounds) represents a highly diverse set of chemotypes designed from in-house research experience, while the Advanced Collection (>278,000 compounds) is intended for lead discovery. The latter set of compounds has been designed according to lead-like properties (MW ≤ 350, cLogP ≤ 3, and rotB ≤ 7) and/or valuable pharmacophores such as carboxylic, primary amino and amide groups. This database also provides diversity sets derived from the screening collection: a drug-like set (20,160 compounds, Lipinski [21] & Veber’s [36] rules-compliant with no reactive functional groups), a pharmacological diverse set (10,240 drug-like compounds clustered by activities from biologically relevant chemical space) and a 3D diversity set (50,240 compounds from conformational analysis and shape clustering of HTS collection) as well as targeted libraries (CNS, antibacterial, ion channel, kinase and Lipid GPCR libraries), fragment libraries (general, golden -multi-purpose fragment tool library-, covalent, sp3-rich, PPI, fluorinated and brominated fragments) and Enamine’s REAL database which is a virtual collection of over 2 × 10^7^ structures of various novel compounds that can be successfully synthesized. This collection can be filtered according to specific criteria (diversity, scaffold type, MW, drug-likeness, Lipinski’s rule-based, ADMET properties, etc.). The structure data files from various regularly updated collections of compounds can either be directly downloaded from the webpage in MDL SD (.sdf) or MDL ISIS (.db) formats or obtained by request.

### 2.8. CHEMBRIDGE Database

ChemBridge [41,42] encompasses one million drug-like and lead-like compounds in two non-overlapping collections of respectively 460,000 and 620,000 compounds, that cover different chemical spaces and that can be customized to create diversity libraries, targeted libraries (KINASet, CNS-Set, and IONSet libraries) and fragment libraries, which can be purchased upon request. In particular, the EXPRESS-Pick library intends to embrace broad chemical spaces and offers different classes of compounds of high diversity with analogues to assist in structure–activity relationship (SAR) studies, and the CORE library undertakes non-covered chemical spaces by other libraries and is enhanced with a large number of sp3-rich scaffolds. The screening libraries can be downloaded as MDL SD (.sdf) or MDL ISIS (.db) files.

## 3. Virtual Screening Protocols and Techniques

**Figure 1:**
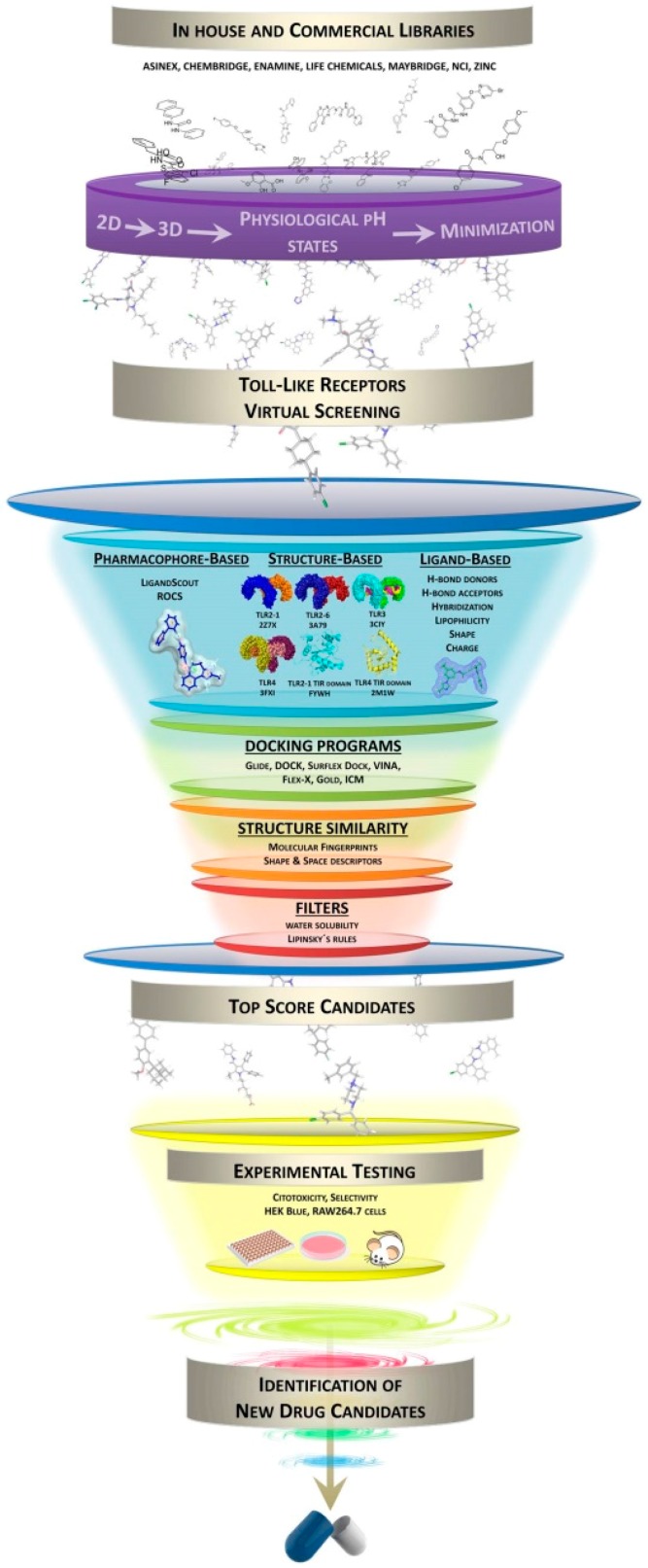
Summary of the virtual screening (VS) protocols applied for the search for novel Toll-like receptors (TLR) modulators: access to databases and preparation/filtering of small-molecules; pharmacophore generation; docking calculations; selection of candidates; experimental testing, and final identification of drug candidates.

General strategies for a VS protocol include several steps that are summarized in Figure 1. The availability of the 3D coordinates of the target is mandatory, either from X-ray crystallography, NMR or homology modeling. Prior knowledge about the ligand binding site may help in the identification of proper binders although, in some approaches, the search for novel binding pockets can be an additional interesting—and challenging—element in the drug discovery process. In this review, we will not focus on these aspects but rather describe the computational protocols employed, so far, to perform virtual screening on Toll-like receptors. We will go over database processing, pharmacophore generation and, finally, docking tools successfully used for VS in TLRs (Table 1).

### 3.1. Database Processing and Inclusion of Decoys

Database processing constitutes a fundamental step in VS approaches. It is crucial to generate the proper chemical library, with the adequate geometries, ionization states, conformations, tautomers, etc. Furthermore, it is very important to discard any molecule that will not be a good candidate in the further steps of the VS study in relation to the particular system on hand. A good database processing will assure a rigorous and well-conducted virtual screening, as well as avoiding computational costs and identification of unsuitable drug candidates.

#### 3.1.1. LigPrep

LigPrep [43], a software created by Schrödinger LLC, is a collection of tools designed to prepare high quality, all-atom 3D structures for large numbers of drug-like molecules, starting from 2D or 3D structures. LigPrep starts by converting the input structure files to Maestro [44] format. The LigPrep process consists of a series of steps that perform conversions, apply corrections to the structures, generate variations on the structures, eliminate unwanted structures, and optimize the geometry. LigPrep produces a single low-energy 3D structure with defined chiralities for each processed input structure, and it can also produce a number of structures from each of the starting geometries with varying ionization states, tautomeric forms, stereoisomers, and ring conformations. Additionally, LigPrep offers the option to eliminate molecules from the collection to be screened using various criteria including molecular weight or quantity and types of functional groups composing the molecule.

#### 3.1.2. OMEGA

OMEGA [45] is a software program created by OpenEye Scientific Software (Santa Fe, NM, USA) that generates conformations of molecules. The process used by OMEGA relies on the construction of a database of fragments, from which the molecule will be assembled, and the derivation of a torsion library.

To quickly sum up the process, OMEGA starts by preparing the fragment database by fragmenting a very large collection of compounds into contiguous ring systems and small linear linkers. Then, each fragment undergoes an optimization process in order to generate one or more 3D conformations per fragment. The next step is a torsion sampling library generation process, determining the bonds that may freely rotate, and assigning them a list of possible dihedral angles to each rotatable bond, using SMARTS [46] matching. All torsions are altered by 120 and 180 degrees, and a RMSD calculation is performed. The resulting conformers are placed into a list ranked by energy, and entire structures are assembled by combining the lowest energy set of fragments, and the energy window of the global minimum structure. A final ensemble is selected by sequentially testing the conformers using the RMSD cutoff (user-defined). It is populated up to the user defined maximum ensemble size limit, or until the list of low energy conformers is exhausted.

The third step is the generation of 3D structures, fragmenting the molecular graphs of the conformers in the same manner as the fragment database, and comparing them to each other. The fragments are then assembled into the parent molecule by overlapping fragments using geometric and chemical rules, providing one or a small number of initial conformations for the molecule. Then, a large ensemble of conformations without internal clashes or duplicates resulting from common symmetries is generated, comparing every rotatable bond in the previous conformers to the torsion library and scoring them. Minimization is not performed during the entire process to avoid the production of highly folded conformations not reflective of the one found in solution or bound to a receptor.

#### 3.1.3. AutoDockTools

AutoDockTools (ADT) [47] is the graphical interface implemented within the Python Molecular Viewer to make AutoGrid and AutoDock (both are required to be used) widely accessible tools [47,48,49]. It facilitates the formatting of input molecule files, with a set of methods that guide the user through protonation, calculation of charges, and specification of rotatable bonds in the ligand and the protein. As a brief outline of the preparation process, the ligand is loaded into the graphical interface, and ADT prepares it for AutoDock docking program. Polar hydrogens are added, charges are calculated, and nonpolar hydrogens are merged with the heavier atoms to which they are attached. If the ligand file presents no charges, ADT will compute Gasteiger charges. Then, AutoDock atom types are assigned to each atom. Regarding the ligand preparation for the virtual screening, it is important to consider the flexibility of the ligands. For this purpose, ligand flexibility is assigned in several steps. First, a root atom is chosen, which will act as the fixed position during coordinate transformation in the docking simulation. To find the optimal atom, the number of atoms in each branch is evaluated, and the root atom that minimizes the size of the largest branch is chosen. However, the ligand flexibility can be limited. As a limitation, each step in ADT has to be launched manually, one by one, as well as the preparation of each ligand. However, it is possible, with simple scripts, to do it automatically.

#### 3.1.4. MUBD-Decoymaker

The use of unbiased benchmarking sets is an important aspect of VS validation. Several decoy sets are available and many of them have been used to validate docking scoring functions. In particular, MUBD-Decoymaker [50] has been used for the virtual screening of potential ligands of TLR8 [50]. MUBD-Decoymaker is an unbiased computational method to build benchmarking sets for ligand-based virtual screening. By submitting molecules and decoys to the computational tool, the program will rank them, ensuring chemical diversity of ligands, maintaining the physicochemical similarity between ligands and decoys, making the decoys dissimilar in chemical topology to all ligands to avoid false negatives, and maximizing spatial random distribution of ligands and decoys.

### 3.2. Pharmacophore Generation

Pharmacophore-based strategies have also been used for VS in the context of searching for TLR modulators, LigandScout and ROCS being the most used ones. Other pharmacophore model generation software packages are: Catalyst [51], MOE [52] and Phase [53,54]. When lacking 3D structure of the corresponding TLR, ligand-based pharmacophore generation can be a very useful tool for the identification of putative hits. The combination with structure-based pharmacophores provides more robust models for hit identification.

#### 3.2.1. LigandScout

LigandScout [55] allows the generation of structure- and ligand-based pharmacophore models that can be used in VS. A key characteristic of LigandScout, that distinguishes it from other pharmacophore modeling packages, is that it allows the definition of multiple features per heavy atom. These chemical features are used to perform pairwise alignments of pharmacophores and molecules [50]. For the structure-based approach, the program starts from the macromolecule structure with a bound ligand, either from a co-crystal structure or from a predicted binding pose from docking calculations. The structure-based pharmacophore modeling relies on the automated extraction and interpretation of the key ligand features important for the interaction with the receptor (planar ring detection, assignment of relevant functional group patterns, hybridization state determination, and Kekulé pattern assignment) [55]. Thus, the 3D pharmacophore model contains the chemical features that represent the key interactions with the macromolecule aligned in 3D space, together with a series of excluded volume features defining areas sterically hindered by the macromolecular environment and the shape of the binding site. The model can be used for VS within LigandScout or be exported to other external applications (Catalyst, MOE, and Phase).

Ligand-based pharmacophores can also be created from a set of known active ligands in the absence of a receptor structure. Molecular flexibility of every molecule in the set is taken into account (OMEGA) [55] and each molecule is ranked according to its number of conformations. Chemical features and exclusion volumes spheres are defined and weighted. Intermediate pharmacophore models are generated through the molecular alignment of selected conformations. These intermediate pharmacophore models are ranked using different scoring functions and sequentially aligned to every conformation of every molecule of the set. New intermediate combined feature pharmacophores are generated until at least three common chemical features are identified throughout the whole alignment and interpolation process. Exclusion volume spheres have only been included in the newest version of the program. The directionality of donor/acceptor groups is not taken into account since there may be ambiguity in positioning, given the absence of the receptor structure. LigandScout provides three geometric types of features: vector (H-bond acceptor, H-bond donor, metal binding location for Iron, Magnesium and Zinc atoms), point (hydrophobic interactions, negative ionizable areas, positive ionizable areas, exclusion volume) and plane feature (aromatic ring, plausible π–π or cation–π interactions). The pharmacophore models thus created can then be used in various external VS applications.

Pharmacophore-based virtual screening using LigandScout has been successfully applied to the identification of a novel TLR2 inhibitor in the µM range [56]. A structure-based pharmacophore model of the TLR2 lipopeptide binding site, based on energetically favored potential interactions identified by MIFs (Molecular Interactions Fields) calculations using MOE package [57], was created with the following relevant features: one H-bond acceptor with the NH group of Phe349, one H-bond donor interaction with the carbonyl oxygen of Leu350, and two hydrophobic areas (one covering the region of Phe325, Ile319 and Val348, and a second one around residues Leu328, Leu266, Phe295, Phe284, and Ile314 of the TLR2). Exclusion volumes were added and the 3D pharmacophore was used for virtual screening with LigandScout 3.0 using standard settings against a collection of 2,831,238 commercially available compounds from different vendors. Among the compounds that fitted the pharmacophore, the 150 compounds with the highest pharmacophore fit score were submitted to docking studies using GoldSuite [58]. A subsequent minimization of the binding poses within the binding site was performed with the MMFF94 force field implemented in LigandScout. Five compounds were finally selected for biological studies and one compound (code MolPort-001-796-266, Table 2) was confirmed as TLR2 antagonist. For TLR8 [59], while LigandScout was also used to obtain pharmacophore models within a sophisticated protocol to optimize VS of TLR8 agonists (see Section 4.5).

#### 3.2.2. Rapid Overlay of Chemical Structures (ROCS)

ROCS (Rapid Overlay of Chemical Structures) [60] is a powerful tool for virtual screening which identifies active compounds by shape comparison, based on the idea that molecules have similar shape if their volumes overlay well and that any volume mismatch is a measure of dissimilarity. It is a shape-based superposition method and uses a smooth Gaussian function to represent the molecular volume. The sensitivity is also represented by using hard spheres cut-offs as in many other shape overlay and pharmacophore matching methods. ROCS considers only the heavy atoms of a ligand, ignoring hydrogen atoms. Conformational flexibility is not implemented but it can be taken into account by using precomputed ensembles of conformers (for instance, with OMEGA program [55]).

Although ROCS is primarily a shape-based method, chemical definitions specified by the user can be included into the superposition and similarity analysis process, facilitating the identification of those compounds similar in both shape and chemical features. By default, ROCS compares molecules based purely on their best shape overlap, quantified by their ShapeTanimoto score. Then, the program ranks the database molecules based on their ShapeTanimoto to the query molecule. It was found that adding the so-called “color score” to the ShapeTanimoto score for the appropriate overlap of groups with alike properties (donor, acceptor, hydrophobe, cation, anion, and ring), and then ranking this summed score, the virtual screening performance considerably improved. Cations and anions can be defined according to an implicit pKa model such that the same group had the same protonation state regardless of the protonation state set in the input structure definition. This overlay is then subsequently scored using the sum of ShapeTanimoto for the overlay and the color score (the so-called “combo score”). Customization or target-specific information can be incorporated by adding a term to the color force field file that rewards overlay of specific functional groups. ROCS alignments have also other type of applications: 3D-QSAR, SAR analysis, understanding of scaffold diversity and detection of common binding elements. ROCS alignments to crystallographic conformations have also been useful in pose prediction in the absence of a protein structure [61]. This tool has also been used for a probabilistic framework for structure- and ligand-based virtual screening [62], for use in analog searching and lead hopping [63], and also for comparison of topological, shape and docking methods in virtual screening [64].

In the context of this review, ROCS has been used together with LigandScout, for pharmacophore-based protocols applied to VS on TLRs, specifically on TLR2, TLR7 and TLR8 (see below). In the case of TLR7 [65], since no crystal structure of the target was available, a ligand-based approach was followed by using ROCS to obtain new compounds with similar activity as a query compound. The ZINC database was prepared with OMEGA program. Two query compounds were obtained from two well-known TLR7 ligands. The 3D ligand-based VS was carried out with the prepared ZINC database and the compounds were ranked with the “TanimotoCombo” score (considering shape and atom type). Six new compounds with three new chemical scaffolds were identified as TLR7 antagonists with activities within the µM range (Table 2). In the case of TLR8 [59], in order to generate ROCS queries, the authors performed an alignment of six crystal ligands as initial molecules. The results were ranked using the “ShapeTanimoto” and the “TanimotoCombo” scores. Ligand-based VS in TLR2 has also been performed with ROCS [56]. Using this approach, several novel TLR2 antagonists were identified in the low-µM range. A shape- and feature-based search was run using three known TLR2 modulators as separate queries against the open NCI database using ROCS default settings. The resulting hits with the highest shape and feature overlap were selected for biological testing where four compounds showed inhibitory activity. Two of these four confirmed antagonists were used as query along with two of the initial queries for a second shape- and feature-based search, this time against a larger collection of compounds and an additional docking step using GOLD docking program. After this second search, three additional antagonists were confirmed. More details about this work can be found in Section 4.

### 3.3. Docking Tools for Virtual Screening (VS)

Molecular docking is a well-established method to investigate how a ligand interacts with its receptor. It integrates an automated computer algorithm that determines how a compound may bind in the active site of a target (binding mode and ligand–receptor interactions) and that tries to predict how tightly it binds (prediction of the binding energy), revealing the electrostatic and steric complementarity between the protein and the ligand. Other current challenges in structure-based drug design, outside the scope of this review, include the inclusion of the protein flexibility, active water sites, searching of binding pockets, among others [66,67,68].

Nowadays, most of the docking programs are characterized by (i) the specific method to treat ligand flexibility [69], which can be divided into three categories: systematic methods (incremental construction and conformational search); random or stochastic methods (Monte Carlo, Genetic Algorithms and Tabu search); and simulation methods [70] (molecular dynamics and energy minimization); (ii) the scoring function [69], classified into three categories: (ii.a) force field-based scoring functions [71], where a classic force field is employed to compute the noncovalent ligand-target interactions, such as van der Waals and electrostatic energies (they are often augmented by a GB/SA or PB/SA term in order to account for the solvation effect); (ii.b) empirical scoring function [72,73], where the overall binding free energy is calculated by adding the contributions from several energetic terms, including hydrogen bond (H-bond) interaction and hydrophobic interaction (the weighting factors of all terms are calibrated from a set of known complexes with experimentally determined structures and binding affinities); and (ii.c) knowledge-based (KB) scoring functions [74,75], where the ligand–target interactions are computed as a sum of distance-dependent statistical potentials between the ligand and the target (only the structural information of ligand-target complexes is needed, which is being accumulated rapidly due to structural biology advances).

Here, we briefly outline the most used docking tools for virtual screening in the TLRs field, which are Glide, AutoDock VINA, GOLD, Surflex-dock, FlexX, ICM, and DOCK (Table 1). The reader is referred to the original papers for a detailed account.

#### 3.3.1. Glide

Glide is a commercial docking program provided by Schrödinger [83,84,85]. It uses a hierarchical series of filters to search for possible locations of the ligand in the active-site region of the receptor. It has a systematic method to treat ligand flexibility, with an exhaustive search algorithm. The Glide protocol is intuitive and relies on four steps: the ligands and protein preparation, the receptor grid generation, and the docking process. Before launching the docking step, Glide has to generate a grid that represents the shape and the properties of the receptor, using several different sets of fields that provide progressively more accurate scoring of ligand poses. The grid permits to dock only the relevant region of the receptor, thus saving calculation time.

Regarding the last point, the full docking VS workflow includes three docking stages: HTVS, SP (Standard Precision) and XP (Extra Precision). The first stage performs High Throughput Virtual Screening (HTVS) docking. It is intended for rapid screening of a very large number of ligands and has much more restricted conformational sampling than SP docking. The second stage performs SP docking. It is appropriate for screening ligands of unknown quality in large numbers. The third stage is the XP docking and scoring. It is a more powerful and discriminating procedure using an implementation of a modified and expanded version of the ChemScore scoring function, called GlideScore [84] and categorized as an empirical scoring function. Glide can be used to perform virtual screening, accurate binding mode precision and, furthermore, Glide exhibits excellent docking accuracy and high enrichment across a diverse range of receptor types.

#### 3.3.2. AutoDock VINA

AutoDock VINA (Vina is not AutoDock) is an open-source molecular docking program [86]. It has no graphical interface but it is compatible with MGLTools [87]. However, although MGLTools need other files, such as AutoDock and AutoGrid parameter files (GPF, DPF) and grid map files, VINA does not need them. All it requires is the 3D structures of the molecules to be docked and the specification of the search space including the binding site. One limitation in VINA is that the maximum number of predicted binding poses is limited to 20 per ligand. AutoDock VINA uses a hybrid scoring function. It is inspired by X-score [88] and tuned using the PDBbing [89,90] and extracting empirical information from both the conformational preferences of the receptor-ligand complexes and the experimental affinity measurements. It is both an empirical and a knowledge-based function. Regarding the optimization algorithm, the Iterated Local Search global optimizer is used, and to treat ligand flexibility and optimization, VINA uses a stochastic method with the Iterated Local Search global optimizer [91,92].

#### 3.3.3. GOLD

GOLD (Genetic Optimization for Ligand Docking) [93,94,95] is a commercially available docking program produced from the collaboration between the University of Sheffield, GlaxoSmithKline plc and Cambridge Crystallographic Data Center. It is an automated ligand docking program that uses a stochastic method with a Genetic Algorithm to explore the full range of ligand conformational flexibility with partial flexibility of the protein, and it satisfies the fundamental requirement that the ligand must displace loosely bound water on binding.

In order to address ligand conformational flexibility in the binding pocket, GOLD offers a choice of several scoring functions: GoldScore [94], ChemScore [73], ASP [96,97], ChemPLP [98,99] and also a user-defined score. GoldScore is categorized as a force field-based scoring function. It is the original scoring function provided with GOLD, and has been optimized for the prediction of ligand binding positions rather than binding affinities. It takes into account factors such as H-bonding energy, van der Waals energy, metal interaction and ligand torsion strain. ChemScore is an empirical scoring function which incorporates, contrary to GoldScore, a term (dG) that represents the total free energy variation that occurs upon ligand binding. It also incorporates a protein-ligand atom clash term and an internal energy term. Unlike GoldScore, ChemScore was trained by regression against measured affinity data, although there is no clear indication that it is superior to GoldScore in predicting affinities. ASP (Astex Statistical Potential) is a knowledge-based scoring function that can be compared to PMF [96,100] and DrugScore [75,101]. It uses an atom-atom distance potential derived from a database of protein-ligand complexes. ASP incorporates some ChemScore terms. Finally, ChemPLP is an optimized empirical scoring function. It uses the ChemScore H-bonding term and multiple linear potentials to model van der Waals and repulsive terms. In recent issues, it has been shown that it outperformed the previous scoring functions for both pose prediction and virtual screening purposes [102]. ChemPLP is now the default scoring function in the GOLD recent releases.

#### 3.3.4. Surflex-Dock

Surflex-Dock [103,104] is a commercially available docking program distributed by Tripos and based on an idealized binding site ligand called “protomol” [105] used as a target to generate putative poses of molecules or molecular fragments relying on a molecular similarity method, which are scored using a re-parameterized Hammerhead empirical scoring function [106] with additional negative training data [107]. Like Hammerhead, Surflex-Dock has one mode that uses a systematic incremental construction search approach, to treat ligand flexibility. Additionally, Surflex also uses the whole molecule approach.

#### 3.3.5. FlexX

FlexX [108] is a commercial based docking program provided by BioSolveIT included in the LeadIT package. To handle ligand flexibility, FlexX uses a systematic method with an incremental reconstruction algorithm, where base fragments are identified first, and then, placed into the receptor active site, using a hashing technique. The whole ligand is thus constructed by adding the remaining components step by step selecting each time the optimal partial solution. The scoring function is SCORE1 [109] which is an implemented empirical Böhm function that uses the de novo design program called LUDI [110]. The function takes into account entropic, H-bonding, ionic, electrostatic, aromatic and lipophilic interactions terms.

#### 3.3.6. ICM

ICM (Internal Coordinate Mechanic) is a commercially available docking program provided by MolSoft and based on a stochastic pseudo-Brownian sampling and local minimization [111]. ICM can read, build, convert, refine, analyze and superimpose molecules, plus providing target evaluation to generate 3D models. The ICM docking algorithm, a Metropolis Monte Carlo energy minimization [112], is based on global optimization of the energy function describing the intra-molecular ligand energy and the total interaction energy of the ligand-receptor complex. Conformational sampling is based on the biased probability Monte Carlo (BPMC) procedure [113] which randomly selects a conformation in the internal coordinate space and then moves toward a new random position independent from the previous one but according to a predefined continuous probability distribution to find the global minimum of the energy function.

Two scoring functions (ICM Score and PMF Score) [114,115] based on two diverse approaches of evaluating ligand–receptor interactions are implemented in the ICM docking software. The ICM Score is an empirical scoring function based on calculation of physiochemical properties of the receptor–ligand complex [116]. The ICM Score is calculated as the weighted sum of scores describing the energy terms evaluated during docking simulations (Grid score, H-bond score, electrostatic score, and surface score). The PMF (Potential of Mean Force) Score is a statistical knowledge-based scoring function based on structural information of known protein–ligand complexes [96].

#### 3.3.7. DOCK

DOCK [77,117,118] is a docking software free for academic use, produced at the University of California San Francisco (UCSF). To handle ligand flexibility, DOCK uses a systematic method with an incremental construction algorithm called anchor-and-grow. In this strategy, the largest rigid portion of the ligand (called anchor) is identified and oriented in the active site. The flexible portions of the ligand are then systematically grown from the anchor, clustering at each layer of growth to maximize geometric diversity, until a full molecule is formed. It realizes a superimposition of the ligand onto a negative image of the binding pocket. DOCK uses several scoring functions, the main one being a force field-based scoring function called DOCK 3.5 score, and additional scoring options during minimization, electrostatics calculations, ligand conformational entropy corrections, ligand/receptor desolvation, molecular dynamics simulation capabilities, and other options.

## 4. Virtual Screening in Toll-Like Receptors: TLR2, TLR3, TLR4, TLR7 and TLR8

This section is intended to review the VS studies reported to date in the TLR modulators field. A brief outline about the particular TLR is provided to facilitate the understanding and better follow the work performed. VS protocols and different computational steps are detailed, together with the main results from biological testing. Table 2, Table 3 and Table 4 contain some of the chemical structures of the main TLR modulators identified so far by VS approaches.

### 4.1. Virtual Screening Studies in TLR2

**Figure 2:**
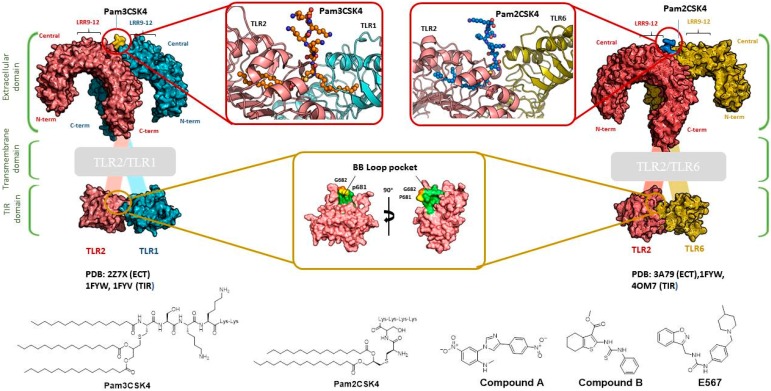
Representations of the 3D structures of TLR2/1 (**left**); and TLR2/6 (**right**) complexes with Pam3CSK4 and Pam2CSK4, respectively. The 2D structure of some compounds mentioned in the text are shown.

TLR2 heterodimerization either with TLR1 or TLR6 mediates specific ligand recognition of bacterial lipopeptides [47]. The X-ray crystallographic structures of both extracellular heterodimers have been resolved assisted by homology modeling in the past few years in complex with the triacylated [119] and diacylated [120] synthetic lipopeptides Pam3CSK4 and Pam2CSK4, respectively (Figure 2). The crystal structure of the TLR2/TLR1 heterodimer [119] with the triacylated lipoprotein revealed that the two ester-linked lipid chains are inserted into the large TLR2 pocket in extended conformation, and the remaining amide-bound lipid chain is inserted into a narrow channel present in TLR1. The binding site is mainly composed of hydrophobic residues from Leucine-rich repeat (LRR) modules 9–12 in both receptors. The peptidic head establishes contacts with polar groups from Phe349 from TLR2, and Gly313 and Gln316 from TLR1. Interestingly, in the case of the (mouse) TLR2-TLR6 heterodimer co-crystalized with the diacylated lipopeptide, the TLR2–lipid interaction and strong PPIs seem to be the prime force for heterodimerization and signaling since the TLR6 channel is shortened by the presence of the bulky side chains from Phe343 and Phe365. A H-bond between the Phe319 (TLR6) backbone and the first peptide bond of the lipopeptide is herein detected.

Regarding the application of VS tool for the finding of novel TLR2 modulators, Zhong et al. [121] report the identification of a natural product-like inhibitor of TLR2/TLR1 heterodimerization (code ZINC12899676, Table 2) following a structure-based VS strategy, through the docking of a collection of natural products and natural product-like compounds from ZINC database (>90,000 compounds) to a TLR2/1 ectodomain model based on the TLR2/TLR1/Pam3CSK4 crystal structure (PDB-ID: 2Z7X). The model was minimized using the BPMC algorithm [113,122], and grid potential maps accounting for hydrogen-bonding, van der Waals, hydrophobic, and electrostatic interactions were calculated. Flexible ligand docking was performed using the virtual library screening module in the ICM-Pro program [123] at the TLR2/TLR1 heterodimeric interface. The 17 best ranked solutions according to the Full ICM Score compounds were selected for biological testing. Among these 17 compounds, compound ZINC12899676 (Table 2) could decrease the secretion of pro-inflammatory cytokines TNF-α and IL-6 in RAW264.7 macrophages stimulated with the most studied TLR2/TLR1 agonist, Pam3CSK4. It showed that it could reduce the secretion of TNF-α by 44% over the concentration range of 0.25 to 4 mM, with an IC_50_ value of ca. 6.1 mM, and the secretion of IL-6 by 56% on the concentration range of 0.25–2 mM, with an IC_50_ value of ca. 1.9 mM, displaying similar potency to the only other TLR2/TLR1 small molecule antagonist reported to date (CU-CPT22) [124,125] with no cytotoxic activity being detected. Compound ZINC12899676 also demonstrated its ability to reduce the phagocytic activity of RAW264.7 cells.

The mechanism of the antagonist activity exhibited by compound ZINC12899676 is proposed to be by displacement of the synthetic lipopeptide Pam3CSK4, as shown by docking studies where two key H-bonds were identified: one between the carbonyl oxygen of the oxalamide motif and the Phe312(TLR1) NH group, and a second one between the amide NH group and the carbonyl CO group of Phe325 from TLR2. Complementary biological and biophysical tests corroborated this possible mechanism of action. A fluorescence polarization assay demonstrated the ability of ZINC12899676 to disrupt Pam3CSK4-mediated TLR1/TLR2 heterodimerization in a dose-dependent manner, with an IC_50_ value of ca. 7.2 mM. An immunoprecipitation assay was used to confirm the inhibitory effect on lipoprotein-induced TLR1/TLR2 heterodimerization exhibiting similar potency to reference compound CU-CPT22. Compound ZINC12899676 could attenuate NF-κB-luciferase reporter assay in RAW 264.7 cells with greater potency than CU-CPT22, and in HEK293T cells transfected with pZERO-TLR1, pCMV-Flag-TLR2 and pNF-κB-Luc, and was able to downregulate IĸBα and IKKα/β phosphorylation and IĸBα expression *in cellulo*.

Other interesting results in this field are the work reported by Murgueitio et al. [56]. The authors report the analysis of TLR2 monomer to predict and locate ligand binding sites using Q-SiteFinder and Site Finder applications in MOE. A subsequent structure-based strategy was followed by centering the VS on the lipopeptide binding site sub-pockets P1-P3. A 3D-pharmacophore model was then constructed using LigandScout [55] revealing a hotspot for H-bond acceptors with the backbone nitrogen atoms from Phe349 and Leu350, and for H-bond donors with the carbonyl oxygen of Leu350. Two hydrophobic areas, defined as HYD1 (Ile319, Phe325, and Val348) and HYD2 (Leu266, Phe284, Phe295, Ile314, and Leu328), were also characterized. This model was validated and used to screen a library of more than 2,800,000 commercially available compounds from different vendors (ASINEX, Life Chemicals, Maybridge, ChemBridge, ENAMINE HTS Collection, and SPECS) with the help of LigandScout.

One hundred and fifty compounds with the highest pharmacophore fit score were docked into the TLR2 binding pocket using GOLD [93,94,95] and, after careful visual inspection, five of them were selected for biological testing on a NF-κB reporter assay in the cell line HEK293-TLR2. Compound with code MolPort-001-796-266 (Table 2) exhibited antagonistic activity, and the IC_50_ value was measured in human monocytes obtaining µM values (decrease in TNF-α production: IC_50_ (µM) TLR2/1 = 28.01 ± 1.23, and IC_50_ (µM) TLR2/6 = 10.91 ± 1.38). Its presumed binding mode was studied by means of docking techniques into the TLR2 binding site, displaying the following key interactions: the nitrogen of the sulfonamide group forms a H-bond with the carbonyl oxygen from Leu350; H-bonds are also formed between the backbone NH groups of Phe349 and Leu350 and the sulfonamide oxygen of the ligand; the chlorine substituent of the sulfamoylbenzamide moiety establishes hydrophobic contacts with Ile319, Phe322, Phe325, Val348, and Phe349 residues located deep inside the TLR2 pocket (Figure 2).

A ligand-based strategy was followed using a shape- and feature-based similarity screening assisted by ROCS and using three reported small-molecule TLR2 signaling modulators (compounds A & B, Figure 2) [31] and E567 (Figure 2) [126] against a NCI compound library of 260,071 compounds. Five hundred hits arose from the VS and, after visual inspection, 39 were selected for biological testing. Out of them, four exhibited antagonist activity (hit rate: 10%): compounds ZINC16769362 and ZINC398557 (that were identified from compound B as query structure) and compounds ZINC1758666 and ZINC585632 (from E567 as query) (Table 2).

The same procedure was repeated using compounds A, B (Figure 2), ZINC16769362, and ZINC585632 (Table 2) as queries, this time against the collection of more than 2,800,000 commercially available compounds used in the structure-based approach. From this procedure, 22 compounds were selected for biological testing and three of them displayed antagonistic activity (Z416323354, MolPort-009-737-181, and MolPort-002-914-354, Table 2). Compounds were also tested for TLR2-specificity and toxicity, and the decrease of pro-inflammatory cytokine TNF-α was evaluated in human monocytes.

Additional computational docking studies of ZINC16769362, which showed the lowest IC_50_, were carried out showing that the ligand is embedded into a narrow sub-pocket at the end of the binding site thus interfering with lipopeptide binding. The binding pose is mainly stabilized by H-bonding between the NH groups of the ligand and the CO groups from Asp305 and Pro306 backbones. Additional hydrophobic contacts are provided by the naphthyl group with lipophilic side chains of the pocket residues. The substitution pattern of the phenyl moiety was shown to be crucial for activity, since compounds with other pattern of substitution were inactive, as well as the presence of aromatic rings, as compounds with aliphatic rings were inactive. Overall, these results shown to be very promising for the identification of several novel TLR2 antagonist with activity in the µM range by using virtual screening techniques.

Mistry et al. [127] have also reported the identification of two novel small molecule inhibitors of TLR2 signaling by targeting a pocket within the so-called BB loop of the TLR2 TIR domain (Figure 2). The TIR domain, located on the cytosolic face of all TLRs and adaptor proteins [128] (in TLR2, MyD88 and TIRAP) has been proven to be key for signaling through the mediation of certain homotypic and heterotypic PPIs [129] that triggers downstream signaling cascades and ends in the production of pro-inflammatory cytokines and chemokines [130]. The crystal structures of human TLR2 and TLR1, as well as the P681H mutant of the TLR2 TIR domain [131] revealed that the BB loop connects strand β-B and helix α-B and sticks out of the structure. The P681H mutation in the BB loop has shown to preclude the recruitment MyD88 and therefore TLR2 signaling.

A pocket within this BB loop of *h*TLR2, formed by 10 residues (Tyr647, Cys673, Asp678, Phe679, Ile680, Lys683, Asp687, Asp688, Asp691, and Ser692) neighboring the highly conserved Pro681 and Gly682 pair, was selected as the target for searching new TLR2 modulators. Flexible ligand docking of a collection of commercially available small molecules and FDA-approved compounds (>1 million compounds) was performed using the DOCK algorithm [132] based on the anchor-and-grow search method [133]. First, a primary docking was performed where each rotatable bond was minimized while created without reminimizing the other bonds, with a minimization of the complete molecule once it was built. The most favored conformation of each molecule in terms of interaction energy was conserved. This resulted in the selection of 50,000 compounds that were subjected to a secondary docking step with an additional simultaneous minimization step of all rotatable bonds against the crystal structure (PDB-ID: 1FYW) and three additional conformations obtained from MD simulations of the protein [134].

The top 1,000 compounds that exhibited the most favorable interaction energies, taking into account every protein conformation, led to the selection of 149 compounds and 20 FDA-approved drugs attending to chemical diversity and physicochemical properties for biological testing in HEK293T-TLR2 transfectants. Among them, compound C29 (Table 2) was able to disrupt both TLR2-TLR1 and TLR2-TLR6 signaling induced by synthetic and bacterial agonist in human cell lines. Pam3CSK4- and Pam2CSK4-induced IL-8 mRNA was decreased by compound C29 in stably transfected HEK-*h*TLR2 in a dose dependent manner, as well as IL-1β gene expression in the human monocytic cell line THP-1. Other effects were not exhibited in other TLR agonist- of TNF-α induced signaling nor cytotoxic effects. This behavior was also observed when HEK-*h*TLR2 and THP-1 cells were stimulated with heat-killed or live Gram-positive and Gram-negative bacteria.

Notwithstanding, C29 only showed activity on TLR2/1 signaling pathway, disrupting only P3C-and *Staphylococcus aureus* lipoteichoic acid- induced IL-1β mRNA in murine macrophages. C29L (*O*-vanillin), a derivative from the imine cleavage of C29 in alkaline conditions (NaOH, 65 µM), displayed similar activity and potency in NF-κB luciferase reporter assay in HEK293T cells and has the advantage of a better water solubility. It was shown to be active both in vitro and in vivo. In this work, Mistry et al. also performed an Alanine scanning mutagenesis of every residue within the BB loop using Y647A as a control mutation as it has been reported to play no role in TLR2 signaling [135]. All 10 BB loop pocket mutants resulted crucial for TRR2/1 signaling but not for TLR2/6 signaling, were mutations C673A, I680A, K683A, and S692A were found to not be needed for TLR2/6 signaling.

### 4.2. Virtual Screening Studies in TLR3

Toll-like receptor 3 (TLR3) is located at the membrane of the endoplasmic reticulum, endosomes, multivesicular bodies, and lysosomes. TLR3 forms a large horseshoe shape that contacts with a neighboring horseshoe, yielding a dimer of two horseshoes. The overall horseshoe-shaped structure of the TLR3 ectodomain is formed by 23 repeating LRRs, ligand-binding domain that is composed of leucine-rich repeats (LRRs) [136]. Some X-ray crystallographic structures are available from mouse (PDB-ID: 3CIG and 3CIY) and from human (PDB-ID: 2AOZ and 1ZIW). TLR3 recognizes specifically dsRNA, and the activation of the receptor induces the secretion of type I interferons and pro-inflammatory cytokines, like a TNF-α, IL-1 and IL-6, triggering immune cell activation and recruitment of the adaptor molecule TRIF *via* TIR domain interaction [137]. In contrast to other TLR ligands, dsRNA signaling occurs *via* MyD88-independent pathways [138]. It has also been reported to recognize synthetic analogue polyinosinic-polycytidylic acid, Poly(I:C) [139]. Therefore, the TLR3/dsRNA complex constitutes an important target in multiples infectious diseases and cancer, as it has been shown to be implicated in several infection models like a herpes simplex encephalitis [140], West Nile disease, phlebovirus, vaccinia and Influenza A [141,142,143,144]. It has also been reported that double-stranded DNA from necrotic cells during inflammation or viral infection activates the signal of TLR3 [145].

Cheng et al. have reported the development of small-molecule probes that exhibited activity as competitive inhibitors of dsRNA binding to TLR3 [146]. The authors performed a VS in the dsRNA binding domain of TLR3 using the ENAMINE drug database. The docking protocol was performed into the dsRNA binding domain of mouse TLR3 (PDB-ID: 3CIY) with Glide program. A HTVS protocol was employed for the first docking and ranking, followed by SP protocol for the top 10,000 compounds. The resultant top 5000 compounds were subsequently docked using the more accurate and computationally intensive XP mode of Glide. First top-ranked 100 compounds were selected and re-ranked by predicted binding energy. The authors finally selected nine hits compounds for evaluation by cell assay of TLR3 activation (ENAMINE codes are: T5528092, T5631009, T5630975, T0519-9149, T5626448, T5643856, T5260630, T55994342, T0505-4844, Table 3).

Most of these nine hits were found to share a structural motif: the chemical structure of a d-amino acid conjugated with an aromatic substituent, thus yielding a new pharmacophore for the TLR3 binding site. To select the best ranked compounds, they took into account different benchmarks: (a) predicted binding energy and spatial complementarity; (b) reasonable chemical structures found in the dsRNA-binding site of TLR3; (c) existence of at least one H-bond between the ligand and one of the dsRNA-recognizing residues on the TLR3 surface (e.g., His539, Asn541, and Ser571); and (d) protonation state and tautomeric form of the ligand had to be acceptable.

A dsRNA, Poly(I:C) was employed to selectively activate TLR3 signaling, resulting in the activation of nitric oxide (NO) synthase and the production of NO in RAW264.7 macrophage cells [147]. They monitored the NO level as an indicator of Poly(I:C)-induced TLR3 activation to evaluate the inhibitory activity. Hit compounds T5626448 and T5260630, both derivatives of d-Phe, were identified with IC_50_ values of 154 and 145 µM, respectively. Different analogues were synthesized and SAR analysis was performed. Finally, only one compound, a T5626448 derivative (compound **4a** in Table 3), was identified as a very potent dose dependent TLR3 antagonist, with a low µM IC_50_ value (3.44 ± 0.41 µM). However, in the case of T5260630 analogues, no significant improvement in the activity was observed, so they only focused on the T5626448 derivative family.

Compound **4a** was also tested against homologous TLRs: TLR1/2, TLR2/6, TLR3, TLR4 and TLR7 using TLR specific ligands, but only TLR3 inhibition was observed. Other different biological assays were performed, finding that compound **4a** did not affect cytochrome P450 CYP3A4, CYP2D6, and CYP2C19 isoforms. Tests on RAW264.7 macrophages were also carried out showing low toxicity, and kinase profiling showed that **4a** demonstrates negligible inhibition activity against a panel of 12 representative kinases. Biophysical tests were also carried out, with a negative control, to demonstrate that **4a** binds to TLR3. Fluorescence anisotropy assay demonstrated that this compound competes with dsRNA for binding to TLR3 with a K_i_ value of 2.96 µM. By an ELISA assay, **4a** was also demonstrated to inhibit the downstream signaling transduction mediated by the formation of the TLR3/ds RNA complex, showing that this compound almost completely abolishes the TLR3-mediated inflammation response at its IC_90_ concentration (27 µM). Finally, the inhibitory effects of TNF-α by compound **4a** at 10 µM were also tested with a result of 60% inhibition, agreeing with the results observed in the NO synthase assay.

### 4.3. Virtual Screening Studies in TLR4

**Figure 3:**
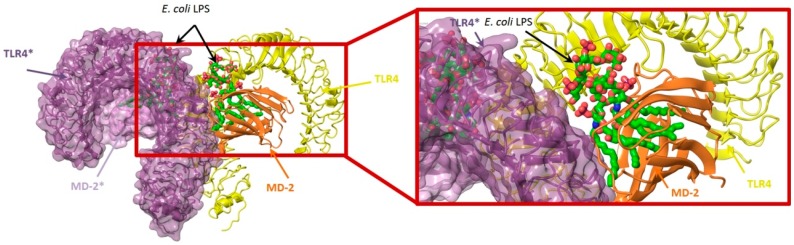
Representation of the X-ray structure of TLR4/MD-2 system (PDB-ID: 3FXI) in complex with *Escherichia coli* LPS. Right view: detail of the LPS (**green**) bound to TLR4 (**yellow**) and MD-2 (**orange**). Partner TLR4*/MD-2* system is represented in violet colors.

Toll-like receptor 4 (TLR4) plays a key physiologic role in host response to Gram-negative bacterial infection [148,149]. An excessively potent and/or prolonged TLR4 response can lead to life-threatening pathology, such as septic shock, inflammation and can also be associated with autoimmune diseases [149]. TLR4 is a trans-membrane protein, being the ectodomain widely studied and believed to be primarily responsible of triggering TLR4/MD-2-dependant innate immune response by dimerization upon LPS binding. It is well established that, to be activated, TLR4 needs to form a heterodimeric complex with its accessory protein myeloid differentiation factor 2 (MD-2) [150,151]. This TLR4/MD-2 complex will bind to lipopolysaccharide (LPS), a membrane constituent of Gram-negative bacteria, which leads to the dimerization of two TLR4/MD-2 complexes, and the final activation of the innate immune system response. As a brief outlook of the interactions, fatty acid chains for the lipid A moiety of the LPS are recognized and inserted into the hydrophobic MD-2 pocket, while the polar moieties (sugars, phosphate groups, and polysaccharidic core) interact with both the rim of MD-2 protein, constituted with polar residues, and some other polar residues of TLR4 (Figure 3) [152]. These interactions will lead to and stabilize the heterodimerization of the complex, triggering an intracellular cascade of events leading to the immune response activation. Therefore, TLR4 has attracted much attention for the discovery of new modulators with important applications in biomedicine [153].

In the search for novel TLR4 modulators, Yin et al. have applied a computational methodology to the identification of small drug-like inhibitors of TLR4/MD-2 PPIs [154]. The authors have developed a novel in silico screening methodology incorporating molecular mechanics (MM) and implicit solvent methods [154] to evaluate binding free energies, in order to improve affinity prediction accuracy without reducing screening speed. The ENAMINE database collection was screened against the TLR4/MD-2 complex of the crystal structure of the human TLR4 TV3 hybrid-MD-2-Eritoran complex (PDB-ID: 2Z65). The library was clustered to ensure the least possible computational work, while keeping as much of the full chemical diversity of the available library as possible. A combination of Jarvis-Patrick and Li algorithms [155,156] was used; as well as the Tanimoto similarity calculation [27,157,158,159] with Daylight fingerprints in order to measure the distance between the molecules. About 86,000 clusters were isolated. Then, the compounds representing the cluster centroids were taken, and an additional filter that matched the molecular volume to the binding site was applied.

Fast molecular docking for the generation of binding poses and subsequent MD simulations were performed to rank the ligand poses according to their binding affinities. The hits were profiled against a library of 500 representative human proteins as a selectivity filter in order to remove the non-specific inhibitors. Finally, as a proof of concept, the compounds were screened against both TLR4 and MD-2 to validate the strategy [154]. Two compounds, T5342126 and T6071187 (Table 4), were identified as potential TLR4- and MD-2-specific antagonists, respectively, completely abolishing LPS-induced activation of signaling. Their biological activity and selectivity were tested in vitro using Akt1 and nitric oxide in RAW264.7 cells.

In another study, Gobec et al. [160] performed parallel ligand-based and structure-based virtual screenings in order to identify novel TLR4 antagonists targeting the TLR4/MD-2 interface using the crystal structure of the human TLR4 TV3 hybrid-MD-2-Eritoran complex (PDB-ID: 2Z65). For both ligand-based and structure-based virtual screening, they used the ZINC drug-like subset (~11.3 million drug-like compounds) from the ZINC database [24].

Regarding the ligand-based virtual screening, they used the OMEGA software [45] on the compound T5342126 (Table 4), a known TLR4 antagonist [160], to generate five query conformers. ROCS software was then used to compare the database to all query conformers. The single best overlay hits were ranked according to the TanimotoCombo scoring function [45], considering similarities in the molecular shape and color of atom types. Thereby, five compounds were identified (ZINC51408124, ZINC464832, ZINC26905159, ZINC32525142 and ZINC32524933, Table 4) and evaluated in vitro. Unfortunately, these compounds were either not water soluble, or not active, or presented cytotoxicity on HEK293 cells.

**Figure 4:**
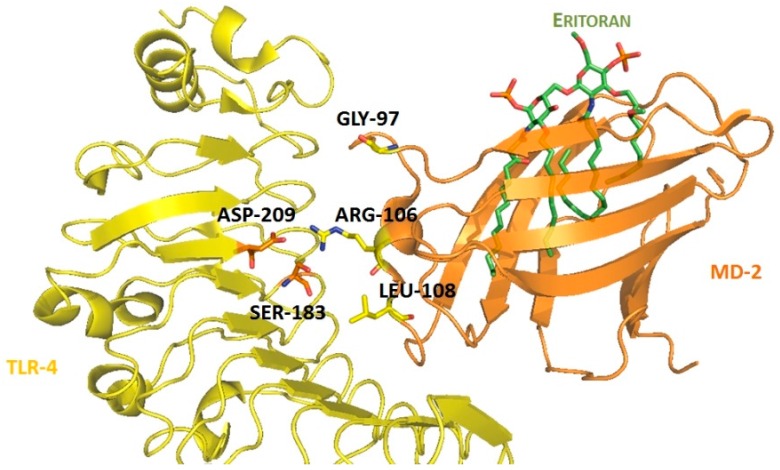
3D structure representation of the extracellular domain of the TLR4/MD-2 complex with Eritoran (PDB-ID: 2Z65) focused on the interaction surfaces between TLR4 (**yellow**), and MD-2 (**orange**). Polar amino-acid residues used to perform the docking procedure are shown in sticks.

For the structure-based virtual screening, before the docking process, they performed an enriching procedure, using ROCS software between the database and T5342126, the query molecule, in order to reduce the number of compound and to enrich it. Two sets of 25,000 compounds each were created: set 1 with the highest shape similarities to T5342126, using ShapeTanimoto algorithm [163], and set 2 with both the highest shape and color (atom type) similarities to T5342126, using the TanimotoCombo algorithm [164]. Both sets were merged and the duplicates were removed, leading to a total of 49,600 unique compounds left. The docking procedure was carried out using FlexX program and the active site was defined as an area of TLR4 within 8 Å around the interacting MD-2 loop (Gly97-Leu108). LeadIT-implemented pharmacophore constraints were performed then in order to keep only the compounds that can form interactions with at least one of the polar amino-acid residues such as Ser183 and Asp209 of TLR4, and Arg106 of MD-2 (Figure 4). At the end of the implementation, 25,750 compounds had been kept, and the docking procedure was performed. The compounds have been finally ranked according to their best scoring conformation using LeadIT score and 40 were selected and assessed in vitro. After the first in vitro assay, only 14 compounds were sufficiently water-soluble, up to 500 µM, and completely non-cytotoxic at 100 µM. Those received further biological evaluation using HEK-Blue^TM^ *h*TLR4 cells, and three compounds with promising antagonistic activities were discovered: ZINC25778142, ZINC49563556 and ZINC3415865.

In another work, Sowdhamini et al. [162] used homology modeling, docking, and virtual screening techniques, in combination with known experimental data, molecular mechanics calculation to identify novel and potential small molecule inhibitors of TRAM-mediated TLR4 signaling. For this purpose, they identified TLR10 TIR dimer as the best model to build the TIR domain of TLR4 as a dimer. Then, they modeled the C-terminal region of the A46 poxviral protein containing the VIPER motif, using the crystal structure of A52 poxviral protein (PDB-ID: 2VVW) as a template. This motif is capable of binding the TIR domain of different adaptor proteins. After having obtained the two models, they performed a two-phase docking for creating reliable models of the complex between the TRAM TIR homology model and the VIPER peptide segment. A virtual screening was then performed on the complex. They used the lead-like and drug-like subsets of the ZINC database, totaling 32 million compounds. The ligands 2D structures were converted into their 3D structure including all possible stereoisomers, tautomers, and ionization states under a pH range of 6–8. Then, the hydrogens were added and the structures were optimized and minimized in LigPrep. The library was preliminary screened based on ADMET properties and reactive functional groups, using Qikprop [165] and Lipinski’s rule of five [21]. The amino acid residues constituting the BB loop (110–122) and alphaC helix (141–154) of the TLR4 TIR domain were selected to generate the receptor grid.

Glide was used for the docking by concatenating the three protocols: HTVS, SP and XP. The top 10% compounds, based on the Glide score, obtained from the HTVS step were retained for the subsequent step. These were re-docked using the SP module. The XP module was used to perform a more extensive docking of the top 10% compounds carried forward from the SP step. Final ranking of the compounds was based on their Glide XP Score. The compounds having similar scaffold were then clustered using CANVAS [166,167] resulting in a pool of 265 chemically diverse structures. These selected compounds were submitted to induce fit docking within the Maestro suite [168,169,170] to restrict the flexibility only into the binding site. For this purpose, they used the Glide SP protocol to generate 2000 poses for each molecule within the binding site. Finally, they inspected the top 20 receptor-ligand poses for each ligand to see if potential interaction between the binding site residues and the ligand atoms were maintained or disrupted upon incorporating flexibility to the residues, and the ligands with more interactions conserved throughout most of the poses were selected. Binding free energy calculations were performed on the top two poses generated during induced-fit docking of each compound. These complexes between the TRAM TIR homology model and each ligand were ranked according to this analysis and a final structural analysis of the ligand/receptor interactions was performed, shortlisting 12 molecules (Table 4). Interestingly, compound ZINC08687988 remained firmly bound in the pocket even after incorporating a considerable degree of conformational flexibility during the MD simulations carried out in the complexes. To date, no further biological testing has been performed yet.

### 4.4. Virtual Screening Studies in TLR7

Toll-like receptor 7 (TLR7) is intracellularly located at the membranes of endosomes, endoplasmic reticulum, multivesicular bodies, and lysosomes [171]. Its function is related to defense against viral infection by recognizing single-stranded RNA (ssRNA) and small-interfering RNA (siRNA) from viruses [172,173], including human immunodeficiency virus, influenza, and vesicular stomatitis virus [174]. The host can also utilize TLR7 to detect RNA released into endolysosomes by phagosomal bacteria. Several synthetic ligands have also been reported to modulate TLR7, such as imidazoquinoline derivatives (resiquimod and imiquimod), and guanine analogues [175]. Also, TLR7 recognizes guanosine- and uridine-rich ssRNA, and synthetic polyuridines act as potent ligands [173]. The development of new antagonist modulators could have important applications for the treatment of autoimmune disorders, like rheumatoid arthritis, Sjogren’s syndrome, and systemic lupus erythematosus [176].

Since no X-ray crystallographic structure of TLR7 is available to date, in order to identify TLR7 modulators, Gobec et al. undertook a ligand-based VS [65]. ROCS was employed to carry out the screening protocol from which six compounds with three novel chemical scaffolds were discovered. The authors employed ZINC database and OMEGA software to prepare the compound library. With the help of ROCS, two query compounds were identified as TLR7 binders: query 1 (imiquimod) and query 2 (1-(4-amino-2-butyl-1*H*-imidazo[4,5-*c*]quinolin-1-yl)-2-methylpropan-2-ol) (Table 3). Imiquimod (query 1) is a TLR7 agonist currently used for topical treatment of genital warts caused by human papillomavirus, actinic keratosis, and superficial basal cell carcinoma [177], and query 2 compound was developed in the last years in a systematic SAR exploration study as the most potent imidazoquinoline with TLR7 agonist activity [178].

From queries 1 and 2, the authors performed parallel VS studies. The results were ranked taking into account the TanimotoCombo score, and the best results from both VS were finally merged. The best 25 ranked compounds were selected and submitted to biological assays, only considering soluble and available compounds. Cytotoxicity tests were performed with HEK-Blue^TM^ *h*TLR7 determined using a propidium-iodide based staining method and none of the compounds showed cytotoxicity at 250 µM. In the subsequent step, the soluble compounds were assayed for TLR7 agonist activity at 250 and 500 µM using the reporter assay but none of the compounds showed any notable agonist activity. Finally, to evaluate the antagonist activity, the compounds were tested using HEK239 cell line co-transfected with *h*TLR7 gene using imiquimod as a control. Six compounds were identified as antagonists at the µM scale containing three novel chemical scaffolds: chromeno[3,4-*d*]imidazole-4-one, 1*H*-imidazo[4,5-*d*]pyridazine-4,7-dione, and 6-amino-9*H*-purine (ZINC codes 12382420, 1667204, 39698, 36416, 4756232, and 8686004, Table 3). The authors also propose a simple and straightforward synthesis of derivatives from the chromeno[3,4*d*]imidazole-4-one scaffold which showed promising TLR7 antagonistic activities.

### 4.5. Virtual Screening Studies in TLR8

Toll-like receptor 8 (TLR8) is an endosomal membrane receptor that recognizes single stranded RNA (ssRNA) from viruses. TLR8 [179] is expressed in monocytes and myeloid dendritic cells [180,181]. TLR8 signaling pathways are mediated by MyD88; this adaptor protein activates NF-κB, IRF-7, and p38 MAPK, resulting in the induction of pro-inflammatory cytokines such as TNF-α, IL-6, IL-1β, IL-12, and antiviral type I interferons. Therefore, TLR8 is a promising target in the development of vaccine adjuvants and anticancer agents [182]. The 3D structure is well-known and six X-Ray crystallographic structures of human TLR8 in complex with six agonists are available (PDB-ID: 3W3J, 3W3K, 3W3N, 3WN4, 4Q8Z, and 4QC0) [183,184,185]. TLR8 consists of an extracellular domain with a horseshoe-shape containing 26 LRR modules, with the ssRNA binding site being very large and flexible. Ligand binding induces reorganization of the pre-organized TLR8 dimer finally enabling downstream signaling processes [183].

To overcome the difficulty of targeting a flexible binding site, Pei et al. [166,167] have performed an enrichment assessment of multiple virtual screening methods, and developed a combined strategy to improve the performance of virtual screening for TLR8 agonists. First, they have created a knowledge-based pharmacophore (KBP) by merging structure-based pharmacophore and previous SAR analysis including furo[2,3-*c*]pyridines, furo[2,3-*c*]quinoles, thiazolo[4,5-*c*]quinolones, 3-*R*-quinolone-2-amine, and C7-methoxycarbonyl-imidazoquines. The combination of the KBP screening with ROCS search was used to improve the efficiency of the virtual screening process. The authors prepared a benchmarking data set merging 13 known active compounds [186,187,188,189], 15 known inactive compounds, and decoys from ZINC database [22]. So, finally, they had 13 actives and 1302 decoys. The benchmarking data set was generated from their recently developed MUBD-Decoymaker protocol [50].

The six TLR8 crystal structures were used to generate SB pharmacophores and shape-based 3D similarity search queries by means of LigandScout software. Eight pharmacophore models were derived with similar backbones in agreement with reported SAR for TLR8 agonists: three hydrophobic centroids, two aromatic rings, one H-bond donor, and one H-bond acceptor. The eight KBPs were used to screen the benchmarking data set in order to select the most robust KBP. The authors selected the so-called “Phar1” as the priority KBP and reserved it for the subsequent antagonist verification.

In order to perform an antagonist verification data set to test the agonist/antagonist selectivity of their selected KBP, 20 reported antagonists were used [178]. For the shape-based 3D similarity search, the authors performed ROCS queries through the alignment of the six ligands from the six crystal structures, using TanimotoShape and TanimotoCombo scores. Among the resulting queries, the so-called “Query4” was further analyzed because of its excellent performance. As an additional step in the protocol, a comparative study was performed with four docking programs: AutoDock VINA, GOLD, Surflex-Dock and Glide. Cross-docking runs were performed with 20 cognate ligands and five dimer TLR8 complexes. Average and median RMSD values were statistically analyzed to determine which program and which crystal structure best matched VS. Taken together, GOLD was identified as the most suitable docking program in conjunction with PDB-ID: 3W3J for the VS evaluation of the protocol.

Finally, the selected pharmacophore “Phar1” was combined with the ROCS “Query4” in different ways to get to the best performance as VS strategy for TLR8 agonists. Final docking with GOLD and PDB-ID: 3W3J, led to the screening of seven compounds, being three of them known active ligands as TLR8 agonists. The authors conclude that this “Phar1_Q4_Gold” strategy was proved to be a promising practice for the identification of novel TLR8 agonists. Indeed, this computational effort can be of help for the design of efficient VS strategies in other TLRs.

## 5. Conclusions

Each designed virtual screening protocol internalizes the knowledge and intuition of the researcher devoted to the work, making each of these approaches almost unique. This review intends to summarize the recently reported (successful) efforts in the search for novel chemical entities with drug-like properties for TLR modulation by means of VS techniques. This work can serve as inspiration for further optimization studies, as well as for fostering this active research field seeking novel drugs for the treatment of severe diseases in which TLR modulation has emerged as a novel therapeutic strategy. Also, this review provides a descriptive overview of the main databases and computational techniques employed for VS approaches.

## Figures and Tables

**Table 1:** Overview of the docking programs employed for virtual screening (VS) in Toll-like receptors (TLRs) mentioned in this review. ST: Stochastic; SYS: Systematic; E: Empirical; FF-based: Force field based; K-based: Knowledge-based.

Program	Ligand Flexibility Method		Scoring Function		Society	
	Type	Algorithm	Type	Name	Name (Availability)	Website
AutoDock VINA	ST	Iterated Local Search global optimizer	Hybrid E/K-based	-	The Scripps Research Institute, la Jolla (Free)	[76]
DOCK	SYS	Incremental construction Anchor-and-Grow Algorithm	FF-based	DOCK 3.5 score	University of California San Francisco (Free)	[77]
FlexX	SYS	Incremental Reconstruction Algorithm	E	SCORE1	BioSolveIT (Commercial)	[78]
Glide	SYS	Exhaustive Search Algorithm	E	GlideScore	Schrödinger (Commercial)	[79]
GOLD	ST	Genetic Algorithm	FF_based E K-based E	GoldScore ChemScore ASP ChemPLP	University of Sheffield, GlaxoSmithKline plc and CCDC (Commercial)	[80]
ICM	ST	Pseudo-Brownian sampling and local minimization	E K-based	ICMScore PMF	MolSoft (Commercial)	[81]
Surflex-Dock	SYS	Incremental Reconstruction Algorithm Whole Molecule Approach	E	Re-parameterized Hammerhead	Tripos (Commercial)	[82]

**Table 2:** 2D Chemical structure of TLR2 modulators identified by VS techniques and mentioned in this review. The database codes are provided. ^a^ MolPort is a supplier of chemicals included in several VS databases (www.molport.com).

TLR2/TLR1	TLR2/TLR6
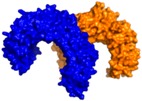	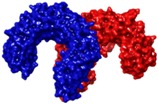
3D structure from PDB-ID 2Z7X	3D structure from PDB-ID 3A79
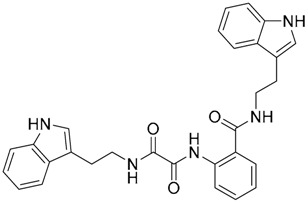	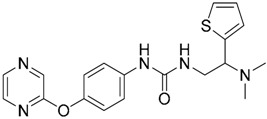
ZINC: ZINC12899676 [121] TLR2-TLR1 heterodimerization inhibitor	ENAMINE: Z416323354 [56] MolPort ^a^: MolPort-009-315-475 TLR2/1 & TLR2/6 inhibitor
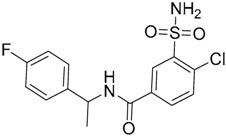	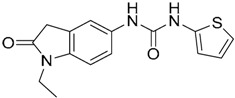
MolPort ^a^: Molport-001-796-266 [56] TLR2/1 & TLR2/6 inhibitor	MolPort ^a^: MolPort-009-737-181 [56] TLR2/1 & TLR2/6 inhibitor with a decrease of cell viability
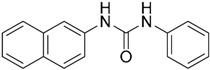	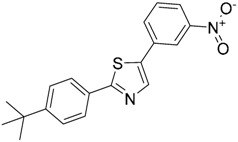
ZINC: ZINC1676936 [56] NCI: Plated 2007: 44661TLR2/1 & TLR2/6 inhibitor	MolPort ^a^: MolPort-002-914-354 [56] TLR2/1 & TLR2/6 inhibitor
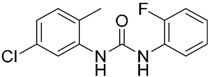	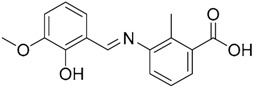
ZINC: ZINC398557 [56] NCI: Plated 2007: 205636 MolPort ^a^: MolPort-001-835-401 TLR2/1 & TLR2/6 inhibitor	C29 [127] TLR2 TIR domain inhibitor
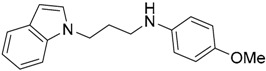	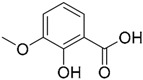
ZINC: ZINC1758666 [56] NCI: Plated 2007: 17379 TLR2/1 & TLR2/6 inhibitor	C29L (*O*-vanillin) [127] TLR2 TIR domain inhibitor
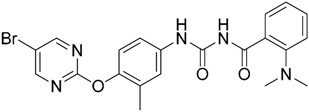	
ZINC: ZINC585632 [56] TLR2/1 & TLR2/6 inhibitor	

**Table 3:** 2D Chemical structure of TLR3 and TLR7 modulators identified by VS techniques and mentioned in this review. The database codes are provided.

TLR3		TLR7	
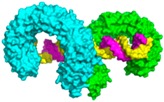		No X-ray crystallographic structure available	
3D structure from PDB-ID 3CIY		-	
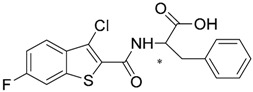	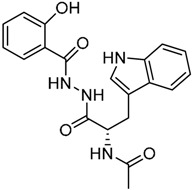	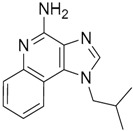	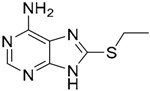
(R) Compound **4a** [146] TLR3 inhibitor	ENAMINE: T5528092 [146] TLR3 inhibitor	Query 1 (Imiquimod) [65]	ZINC: ZINC1667204 [65] TLR7 inhibitor
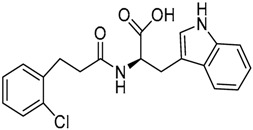	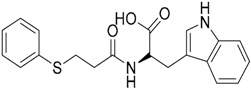	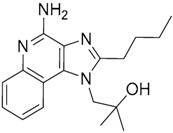	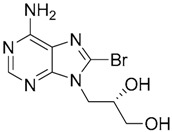
T5631009 [146] TLR3 inhibitor	ENAMINE: T5630975 [146] TLR3 inhibitor	Query 2 [65]	ZINC: ZINC39698 [65] TLR7 inhibitor
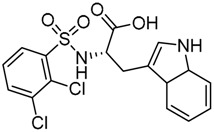	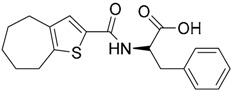	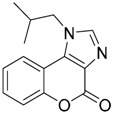	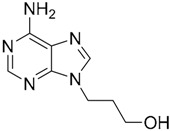
T0519-9149 [146] TLR3 inhibitor	ENAMINE: T5626448 [146] TLR3 inhibitor	ZINC: ZINC12382420 [65] TLR7 inhibitor	ZINC: ZINC36416 [65] TLR7 inhibitor
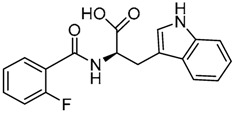	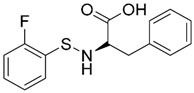	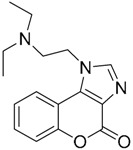	
ENAMINE: T5643856 [146] TLR3 inhibitor	ENAMINE: T5260630 [146] TLR3 inhibitor	ZINC: ZINC4756232 [65] TLR7 inhibitor	
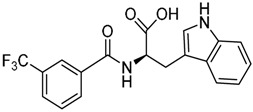	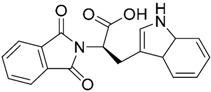	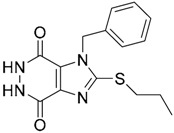	
ENAMINE: T55994342 [146] TLR3 inhibitor	ENAMINE: T0505-4844 [146] TLR3 inhibitor	ZINC: ZINC8686004 [65] TLR7 inhibitor	

**Table 4:** 2D Chemical structure of TLR4 modulators identified by VS techniques and mentioned in this review. The database codes are provided.

TLR4		
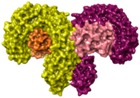		
3D structure from PDB-ID 3FXI		
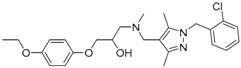	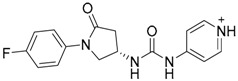	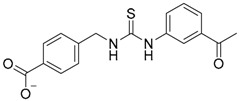
ENAMINE: T5342126 [161] TLR4 inhibitor	ZINC: ZINC04272679 [162] Predicted TLR4 inhibitor	ZINC: ZINC00611718 [162] Predicted TLR4 inhibitor
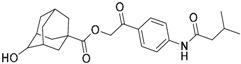	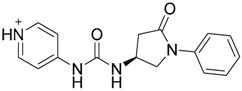	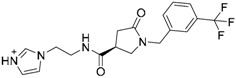
ENAMINE: T6071187 [161] MD-2 inhibitor	ZINC: ZINC04272561 [162] Predicted TLR4 inhibitor	ZINC: ZINC48141941 [162] Predicted TLR4 inhibitor
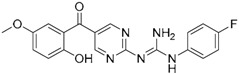	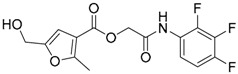	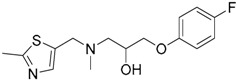
ENAMINE: T5339238 ZINC: ZINC25778142 [160] TLR4 inhibitor	ZINC: ZINC09535665 [162] Predicted TLR4 inhibitor	ENAMINE: T6969316 ZINC: ZINC51408124 [160] TLR4 activity not determined (solubility problems)
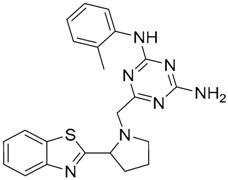	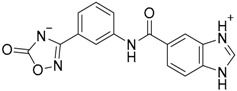	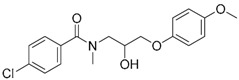
ENAMINE: T5458371 ZINC: ZINC49563556 [160] TLR4 inhibitor	ZINC: ZINC70039563 [162] Predicted TLR4 inhibitor	ZINC: ZINC464832 [160] TLR4 activity not determined (cytotoxicity on HEK293 cells)
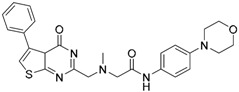	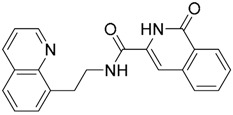	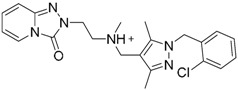
ENAMINE: T5315798 ZINC: ZINC3415865 [160] TLR4 inhibitor	ZINC: ZINC29450369 [162] Predicted TLR4 inhibitor	ENAMINE: T6417643 ZINC: ZINC26905159 [160] Predicted TLR4 inhibitor but not active
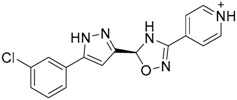	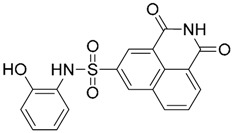	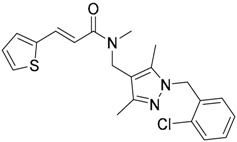
ZINC: ZINC64951618 [162] Predicted TLR4 inhibitor	ZINC: ZINC41124663 [162] Predicted TLR4 inhibitor	ENAMINE: T6280209 ZINC: ZINC32525142 [160] Predicted TLR4 inhibitor but not active
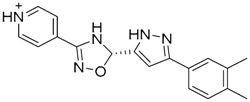	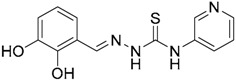	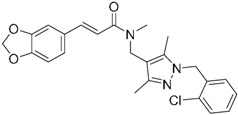
ZINC: ZINC64951738 [162] Predicted TLR4 inhibitor	ZINC: ZINC08687988 [162] Predicted TLR4 inhibitor	ENAMINE: T6279749 ZINC: ZINC32524933 [160] Predicted TLR4 inhibitor but not active
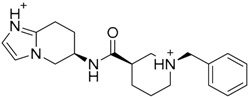		
ZINC: ZINC72278680 [162] Predicted TLR4 inhibitor		

## Abbreviations

2D	Two-Dimensional
3D	Three-Dimensional
3D-QSAR	Three-Dimensional Quantitative Structure-Activity Relationships
ADMET	Absorption-Distribution-Metabolism-Excretion-Toxicity
AIDS	Acquired Immune Deficiency Syndrome
Akt1	Serine-Threonine Protein Kinase 1
BPMC	Biased Probability Monte Carlo
cLogP	Calculated logP
CNS	Central Nervous System
dsRNA	Double-Stranded RNA
ELISA	Enzyme-Linked Immuno-Sorbent Assay
EP	Extra Precision
FDA	Food and Drug Administration
GB/SA	Generalized Born/Surface Area
GPCR	G Protein–Coupled Receptor
HEK	Human Embryonic Kidney cells
*h*TLR	Human Toll-Like Receptor
H-bond	Hydrogen bond
HTS	High Throughput Screening
HTVS	High Throughput Virtual Screening
KB	Knowledge-based
KBP	Knowledge-based Pharmacophore
ICM	Internal Coordinate Mechanics
IL	Interleukin
LPS	Lipopolysaccharide
LRR	Leucine-Rich Repeat
MD Simulations	Molecular Dynamic Simulations
MD-2	Myeloid Differentiation protein 2
MIF	Molecular Interaction Field
MM	Molecular Mechanics
mRNA	Messenger RNA
MW	Molecular Weight
MyD88	Myeloid Differentiation primary response gene 88
NCI	National Cancer Institute
NF-κB-luciferase	Nuclear Factor-κB luciferase
NIH	National Institutes of Health
NMR	Nuclear Magnetic Resonance
NO	Nitric oxide
PAMP	Pathogen-Associated Molecular Pattern
PB/SA	Poisson-Boltzmann/Surface Area
PDB-ID	Protein Data Bank Identification code
PMF	Potential of Mean Force
Poly(I:C)	Polyinosinic-polycytidylic acid
PPI	Protein-Protein Interaction
pZERO-TLR1	cloning plasmid for the expression of TLR1 TIR-deleted genes
RAW264.7	Cell Line murine macrophage from blood
RMSD	Root-Mean-Square Deviation
RNA	RiboNucleic Acid
rotB	Rotatable Bonds
Ro3	Rule of Three
SAR	Structure-Activity Relationship
SDF format	Structure Data File format
siRNA	Small Interfering RNA
SP	Standard Precision
SPR	Surface Plasmon Resonance
ssRNA	Single-Stranded RNA
THP-1	Monocyte from human blood
TIR	Toll/Interleukin-1 Receptor
TIRAP	Toll-Interleukin 1 Receptor domain containing Adaptor Protein
TLR	Toll-Like Receptor
TNF-α	Tumor Necrosis Factor α
TRAM	Translocating Chain–Associated Membrane
UCSF	University of California-San Francisco
VIPER	Viral Inhibitory Peptide of TLR4
VS	Virtual Screening
XP	Extra-precision
